# Total knee arthroplasty at 15–17 years: Does implant design affect outcome?

**DOI:** 10.1007/s00264-013-2231-8

**Published:** 2013-12-18

**Authors:** Jan Victor, Stijn Ghijselings, Farhad Tajdar, Geert Van Damme, Patrick Deprez, Nele Arnout, Catherine Van Der Straeten

**Affiliations:** 1 Department of Orthopaedics and Traumatology, Ghent University Hospital, De Pintelaan 185, 9000 Ghent, Belgium; 2 Department of Orthopaedics, Hospital St-Lucas, St.Lucaslaan 29, 8310 Bruges,, Belgium

**Keywords:** Total knee arthroplasty, Long-term outcome, Implant design, Polyethylene thickness

## Abstract

**Purpose:**

A study was conducted to compare minimum 15-year survivorship and outcome of the Genesis I and II implants for total knee arthroplasty (TKA).

**Methods:**

We retrospectively reviewed 245 consecutive TKA implanted between January 1995 and October 1997. Genesis I was implanted in 156 knees and Genesis II in 89 knees.

**Results:**

At 15–17 years, 75 patients (31 %) had died, 28 patients (11 %) were lost to follow-up and 11 TKA were revised (4.6 %), including ten Genesis I (6.4 %) and one Genesis II (1.1 %); 131 TKA (53 %) were available for follow-up. Cumulative survivorship was 92.4 % at 15.7 years. Survival in patients <69 years at surgery was lower (88.0 %) compared with patients ≥69 years (98.5 %;* p* = 0.023). In patients <69 years, Genesis I survival (84.3 %) was worse compared with Genesis II (97.1 %) (*p* = 0.018). Polyethylene (PE) Insert thickness ≤11 mm had significantly better survivorship (97.1 %) compared with PE >11 mm (56.7 %) (*p* < 0.0001)

**Conclusions:**

At a minimum of 15 years, the overall (92.4 %) survivorship of Genesis TKA was good, with excellent (98.1 %) survivorship of the Genesis II design. Revision rates were higher with Genesis I in the younger age group and with insert thickness >11 mm, possibly due to longer shelf life of less frequently used sizes.

## Introduction

Total knee arthroplasty (TKA) is a common orthopaedic procedure performed with increasing frequency [1]. Outcome is described as the function of several variables, including survival [2], kinematics [3], function [4] and patient satisfaction [5]. None of these approaches covers the full spectrum of reality. Historic papers reporting long-term results are still used as benchmarks in the orthopaedic literature [6, 7] but often deal with obsolete implants and outdated patient selection criteria, large dropout numbers and changes in patient function and activity over 15–20 years. The current patient profile for TKA in terms of age and disease progression is quite different from historic publications [8]. In the United States, the demand for primary TKA among patients <65 years is predicted to exceed 50 % of TKA patients by 2016 [8]. For arthritic patients with 20–40 years remaining life expectancy, reliable long-term follow-up data on TKA are a sound basis for decision making. Therefore, studies on longer-term follow-up after TKA performed in the past decade [7, 9–18] deserve further attention, despite the logistic problems and limitations. Besides, reliable long-term data on implants still in use can serve to evaluate quality and performance of new devices.

In this study, we analysed two different TKA designs: Genesis I and Genesis II (Smith & Nephew, Memphis, TN, USA) at 15–17 years follow-up. Genesis I is no longer commonly implanted, whereas Genesis II is still widely used. Despite the similarity in name, the two implants have different geometry. Genesis I was one of the first modular implants with a built-up module to convert the device from a cruciate-retaining (CR) to a posterior stabilised (PS) design by resecting an additional extra 4 mm from the distal femur on top of the regular 9-mm resection. The polyethylene (PE) post was tall, and accommodation of the femoral box required significant bone removal. In comparison, the Genesis II implant has a nonmodular, distinct CR and PS component, with a more rounded and less prominent post. Also, tibial coverage and the trochlear groove on the femoral component are more anatomic. Hence our research questions were: (1) What is the survivorship and clinical outcome of a consecutive series of TKA at 15–17 years of follow-up? (2) Is there a difference in outcome between the two TKA designs? (3) Can determinant implant and patient factors be established?

## Patients and methods

Between January 1995 and October 1997, 245 consecutive primary TKA were performed on 220 patients by a single surgeon (JV). Cohort 1 consisted of 156 Genesis I knees and cohort 2 of 89 Genesis II knees. Patients were operated through a standard anteromedial approach. The patella was everted and resurfaced in all cases. Femoral alignment was guided by an intramedullary rod and tibial alignment by an extramedullary rod. Knees were implanted with a measured resection technique and standard instrumentation. The choice for a CR or PS implant was made after assessment of deformity and ligament status. All tibial and patellar components were cemented. Femoral fixation included both uncemented and cemented cases.

**Fig. 1:**
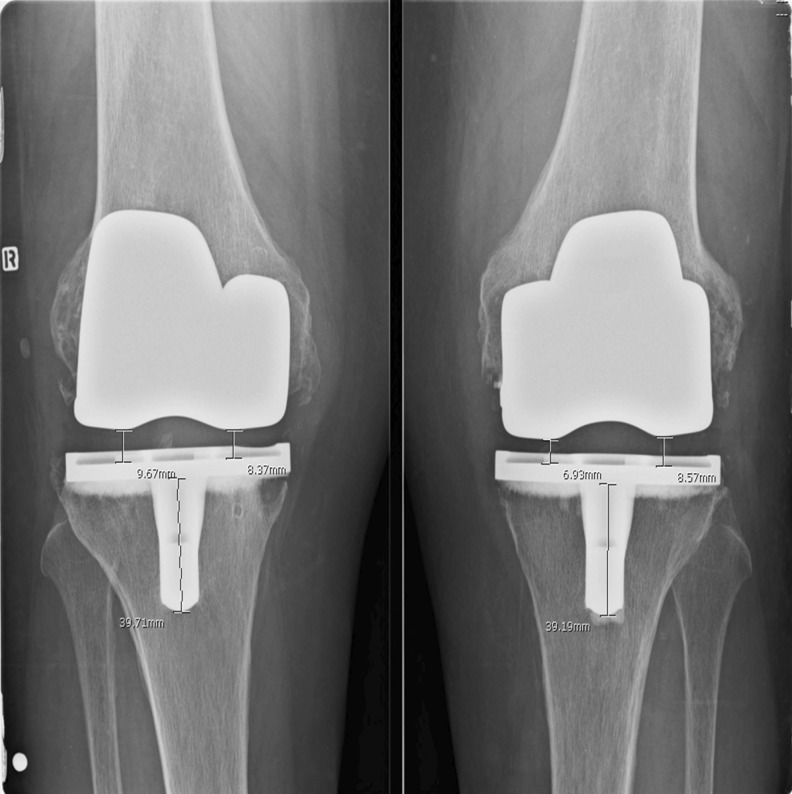
Polyethylene (PE) thickness of medial and lateral compartments measured on anteroposterior radiographs. The estimated amount of linear wear was calculated by deducting the measured thickness, corrected for magnification, from the initial PE thickness, as disclosed by the manufacturer

Clinical and radiographic data were documented in a prospective database. Pre-operative and six week postoperative imaging included full-leg, standing X-rays and standing anteroposterior (AP), lateral and skyline views. At 15–17 years of follow-up, patients were examined by an independent study nurse. Range of motion (ROM), stability and swelling were noted. Knee Society Score (KSS) [19] and Knee Injury and Osteoarthritis Outcome Score (KOOS) [20] were computed. Radiolucent lines were documented on AP and lateral views, according to the Knee Society scoring system [21]. Alignment was measured on full-leg views, represented as the hip-knee-ankle (HKA) angle (angle formed between the tibial and femoral mechanical axes in the coronal plane). PE thickness was measured on AP views of the medial and lateral compartment (Fig. 1). The estimated amount of linear wear was calculated by deducting the measured thickness, corrected for magnification, from the initial PE thickness, as disclosed by the manufacturer.

### Statistical analysis

Data analysis was performed using IBM-SPSS Statistics 21 (SPSS, an IBM Company, Chicago, IL, USA). Level of statistical significance was 0.05. Research questions were examined as follows: (1) Kaplan-Meier survivorship analysis was performed for the total cohort; clinical and radiographic outcome were analysed for the nonrevised knees available at the 15- to 17-year follow-up. (2) Comparison of survival of the two TKA designs was performed using the log rank (Mantel-Cox) test. (3) In order to establish determinant patient and implant factors, subanalysis was performed by gender, age at surgery, body mass index (BMI), femoral fixation mode and PE insert thickness using parametric or nonparametric tests, as appropriate. Log-rank test was used to establish the difference in implant survivorship between subgroups.

## Results

Pre-operative demographic distribution of the two cohorts was comparable in terms of gender, BMI, clinical scores, varus/valgus distribution and amount of pre-operative coronal deformity (*p* > 0.1) (Table 1). Mean age at surgery was significantly lower in the Genesis II cohort (*p* = 0.016). Distribution of femoral component fixation mode and PE insert thickness was similar.

**Table 1:** Demographics of the study cohort and group comparison

	Genesis I	Genesis II	*P* value
Number	156	89	
Gender	M 26.9 %, F 73.1 %	M 25 %, F 75 %	0.417
Age at surgery	Mean 69.3 years (SD 9.45) Median 69.4 years (27–89)	Mean 66.0 years (SD 9.61) Median 67.9 years (27–84)	0.016
BMI	Mean 28.5 (SD 4.77) Median 27.5 (18.7–42.3)	Mean 28.4 (SD 5.03) Median 28.7 (17.5–42.5)	0.811
Femoral component	Cemented 75 (48 %) Uncemented 81 (52 %)	Cemented 42 (47 %) Uncemented 47 (53 %)	0.894
Insert size	Median 10 mm 10 (83 %); 12 (15 %); 15 (2 %)	Median 11 mm 9 (41 %); 11 (51 %); 13 (7 %); 15 (1 %)	0.956
Varus/valgus	98/48	62/25	0.172

*BMI* body mass index, *SD* standard deviation

**Fig. 2:**
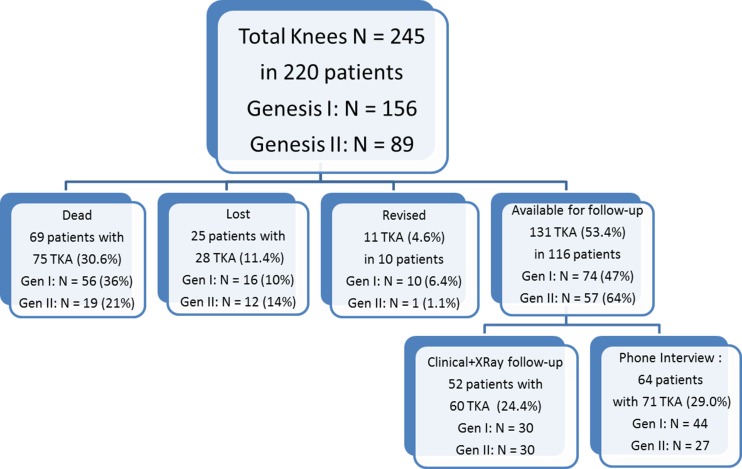
All patients: dead, lost, revised and available for 15–17 years’ follow-up in the global population and in the Genesis I (*Gen I*) and Genesis II (*Gen II*) cohorts

At 15–17 years, 69 patients with 75 TKA (30.6 %) had died, and 25 patients with 28 TKA (11.4 %) were lost to follow-up (Fig. 2). Overall, mean follow-up was 11.0 (0.5–16) years. In all but two lost patients, files indicated an uneventful course at last follow-up. One patient (Genesis I, PS), lost at 11 years, complained of mild pain. He had undergone TKA after a complex tibial fracture. The tibial component displayed 4° varus without radiolucent lines. The other symptomatic patient (Genesis II, PS) was lost after three years, with mild complaints related to the extensor mechanism. Patellar tracking and fixation was fine. All patients in the lost cohort would presently be at least 80 years old (mean age 87, range 80–105), hence, most are probably deceased. Eleven TKA (in ten patients) were revised, including ten Genesis I for infection (1), recurrent dislocation (1), femoral component loosening (1), patella loosening (2) or PE wear (5), and one Genesis II for PE wear (Table 2).

**Table 2:** Details of revised cases of Generation (Gen) I and II total knee arthroplasty (TKA)

Patient		Age at surgery	BMI	TKA type	Constraint/ fixation	Insert size	PE sterilisation	Revised at	Failure mode
1	F	66	25	Gen I	PS/C	12	Gamma	1 year	Infection
2	F	67	36	Gen I	PS/C	12	Gamma	1 year	Dislocation
3	F	64	35	Gen I	CR/UC	10	EtO	9 years	Patella loosening
4	M	63	27	Gen I	PS/C	15	Gamma	10 years	Patella loosening
5	F	68	25	Gen I	CR/UC	12	Gamma	12 years	PE wear /instability
6	M	64	27	Gen I	PS/C	15	Gamma	13 years	PE wear /infection
7	F	71	41	Gen I	CR/UC	12	EtO	11 years	Femoral loosening
8	F	60	32	Gen I	PS/C	10	EtO	15 years	PE wear
9	F	62	37	Gen I	PS/C	12	Gamma	13 years	PE wear/loosening
		61		Gen I	CR/UC	12	Gamma	15 years	PE wear
10	F	27	30	Gen II	PS/C	11	EtO	15 years	PE wear/Instability

*BMI* body mass index, *PS* posterior stabilized,* CR* cruciate retaining, *C* cemented,* UC* uncemented,* PE* polyethylene,* EtO* ethylene oxide

The remaining 116 patients with 131 TKA (53.4 %) could be contacted for the 15- to 17-year follow-up. In 64 patients who were unfit to come to the clinic because of advanced age (mean 83 years) or disease, data were gathered via a telephone interview (71 TKA). Clinical and radiological examination was obtained in 52 patients (60 TKA) (Fig. 2).

### Outcome at 15–17 years

KSS improved from 39 [standard deviation (SD 16.5)] to 81 (SD 9.2) in Genesis I and from 41 (SD 14.1) to 83 (SD 10.3) in Genesis II. Postoperative KOOS scores for symptoms, pain, daily activity and quality of life were similar in both groups (mean total KOOS Genesis I, 86 (SD 33), Genesis II 89 (SD 40);* p* > 0.1). Radiographs six weeks postoperatively revealed varus deformity (HKA > 3°) in 26 knees (range 4–5.5°) and valgus deformity (HKA < −3°) in 13 knees (range −3.5° to −5°). At 15–17 years postoperatively, 60 TKA (30 in each cohort) were analysed. Radiolucencies of 1 mm were noted in two cases on the tibial side and one case on the femoral side. No patellar radiolucencies were observed. In Genesis I, wear on the medial side averaged 0.76 mm (SD 0.53) versus 0.89 (SD 0.52) on the lateral side; in Genesis II, these values were 0.82 (SD 0.52) and 0.79 (SD 0.50), respectively. There was no difference between groups (*p* > 0.1) .

### Survivorship

**Fig. 3:**
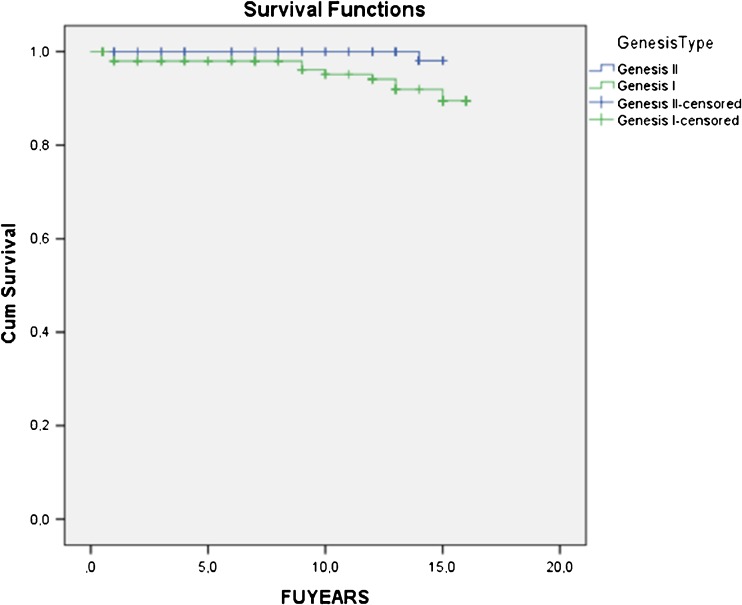
Survivorship in the Genesis I group was 90.1 % at 15.5 years [95 % confidence interval (CI) 88.0 – 92.2) and in the Genesis II group 98.1 % at 15.0 years (95 % CI 97.8 – 98.3). The difference was not statistically significant (Log rank 3.136;* p* = 0.077)

Overall Kaplan-Meier cumulative survivorship was 92.4 % at 15.7 years [95 % confidence interval (CI) 91.0–93.7]. Survivorship in the Genesis I group was 90.1 % at 15.5 years (95 % CI 88.0–92.2) and in Genesis II 98.1 % at 15.0 years (95 % CI 97.8–98.3) (Fig. 3). The difference was not statistically significant (Log rank 3.136;* p* = 0.077).

**Fig. 4:**
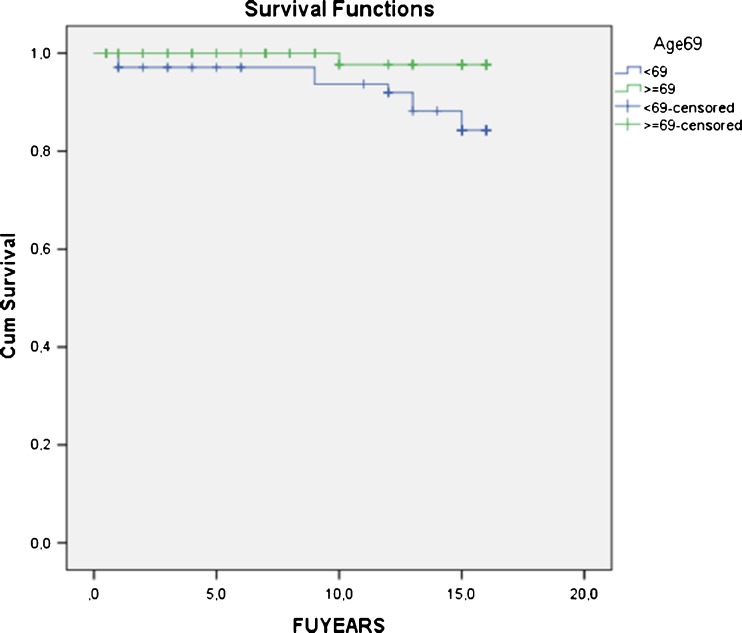
Total knee arthroplasty (TKA) survival in patients <69 years (overall median age) at surgery was significantly worse [88.0 % at 15.4 years; 95 % confidence interval (CI) 85.8 – 90.4] compared with patients ≥69 years at surgery (98.5 % at 15.9 years; 95 % CI 97.4 – 99.6); * p* = 0.023

There was no difference in survivorship between genders (Log rank 0.184; * p* = 0.668) or femoral component fixation (Log rank 1.315; * p* = 0.251). No difference in postoperative coronal alignment between revised and nonrevised patients was observed (* p* < 0.1); although revised patients had a mean BMI of 31.5 (25–41), there was no difference in survivorship between BMI <30 and ≥30 (Log rank 1.403; * p* = 0.236). TKA survival in patients <69 years (overall median age) at surgery was significantly worse (88.0 % at 15.4 years; 95 % CI 85.8–90.4) compared with patients of ≥69 years at surgery (98.5 % at 15.9 years; 95 % CI 97.4–99.6; Log rank 5.135; * p* = 0.023) (Fig. 4). In patients <69 years, survivorship of Genesis II was significantly better (97.1 %; 95 % CI 96.7–97.5) compared with 84.3 % (95 %; CI 80.5–88.1) for Genesis I (Log rank 5.598; * p* = 0.018). The odds ratio (OR) for revision of a Genesis I implant was 12.2 compared with a Genesis II.

**Fig. 5:**
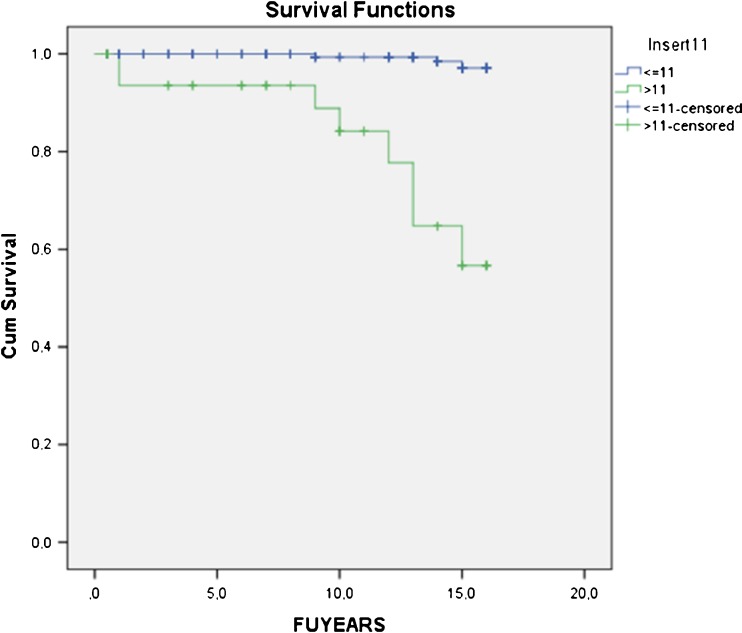
Survival with polyethylene (PE) thickness >11 mm [56.7 % at 14.0 years; 95 % confidence interval (CI) 49.5–62.9] was significantly worse compared with PE thickness ≤11 mm (97.1 % at 15.9 yearsl; 95 % CI 96.5 –97.7); * p* < 0.0001

Analysis of different insert size groups revealed significantly worse implant survival with PE thickness >11 mm (56.7 % at 14.0 years; 95 % CI 49.5–62.9), which were less frequently used (13.9 % of inserts) compared with PE thickness ≤11 mm (97.1 % at 15.9 years; 95 % CI 96.5–97.7) (Log rank 45.657; * p* < 0.0001) (Fig. 5). The OR for revision of a PE insert >11 mm was 35.3.

## Discussion

This study describes the long-term outcome of a consecutive series of 245 TKA in 220 patients consisting of two cohorts of different Genesis implants. Overall, the 92.4 % survivorship at 15.7 years is good, with excellent 98.1 % survivorship of the Genesis II design. The main weakness of the study is the number of dropouts. At 15–17 years, 69 patients had died and 25 were lost to follow-up. Lost patients were >80 years at the time of evaluation; hence, it is logical to assume that many of them had died. In addition, only two of these patients were symptomatic at last follow-up. Advanced age in the majority of patients is a typical phenomenon of long-term joint replacement studies, reducing function scores as a discriminative parameter [17]. Callaghan et al. reported on 26 living patients remaining from an initial cohort of 86 patients. Despite the excellent KSS of 89, the function score dropped to 67. [17]

Minimum 15-year follow-up reports after TKA are not abundant, and several publications deal with implants no longer commonly used. Van Loon et al. followed 102 cemented Kinematic TKA (Howmedica, Rutherford, NJ, USA) at ten to 15 years after surgery and reported 62 % survival at 14 years, with deep infection and wear being the main reasons for revision [10]. Buechel reported 83 % survival of the meniscal-bearing PC-retaining LCS prosthesis (DePuy Warsaw, IN, USA) at 16 years [11]. Ito et al. examined a cohort of 36 Kinematic knees in 25 patients with rheumatoid arthritis and reported 93.7 % survival at 15 years [12]. Dixon et al. found 95.6 % survival at 15 years in 139 PC-retaining press-fit condylar (PFC) prostheses (DePuy), with PE wear as the leading cause for reoperation [13]. Less-favourable results of this implant (85 % survivorship at 15 years) were reported by Duffy et al. due to PE wear and osteolysis from ten years onwards [14]. Baker et al. confirmed these relatively high failure rates at 15-years’ follow-up in 501 primary PFC TKA. Survivorship for cemented implants was 80.7 % and for cementless implants 75.3 % [15]. Paratte et al. studied a cohort of different implants and assessed coronal alignment as a predictive parameter for implant survival. They reported overall survival of 86 % at 15 years [16].

In contrast to the latter findings, we were unable to detect a correlation between postoperative alignment or femoral component fixation and odds for failure. On the other hand, we found a significant difference in survivorship between thinner and thicker PE inserts. Insert sizes >11 mm had a low survival of 56.7 % at 14 years. As these sizes were used less frequently (13.9 % of total), their shelf life may have been longer. Especially in the Genesis I cohort, where gamma radiation in air was still in use as a sterilisation method, this may have caused PE degradation and increased wear, leading to implant failure [22].

Long-term comparative studies of different TKA implant designs are scarce. Kim et al. compared long-term follow-up between two different implants in 108 patients who <51 years at the time of surgery. The patients received an AMK fixed-bearing knee on one side and an LCS mobile-bearing TKA (both DePuy) on the other side. Survivorship at 16.8 year was 95 % for the fixed-bearing knees and 97 % for the mobile-bearing implants [18]. In our comparative study, the Genesis I is no longer commonly in use, but the implant in Genesis II is still widely used. Genesis II TKA with the more anatomic tibial coverage and trochlear design demonstrated superior survival rates compared with Genesis I, but the difference was only statistically significant in the younger age group (<69 at surgery). In the surviving TKA, there was no difference in clinical (KOOS) or radiographic outcome between cohorts. Several reports on the Genesis II implant are available but none with a 15-year follow-up [23–25]. Two prospective, randomized studies compared cruciate-retaining and substituting TKA with this implant and detected no significant difference in function between groups [23, 24]. Bourne et al. [25] reported 98 % survival at a mean of 9.5 (five to 11) years’ follow-up , supporting data obtained in our study. With regard to patient-related factors, in our study, implant survival was worse in women (91.6 % versus 94.8 % in men) and individuals with BMI ≥30 (83.5 % versus 93.5 % with BMI <30); those differences were not statistically significant. Only younger age (<69 years) at surgery significantly increased the revision risk. A recent report from the Finnish Arthroplasty Registry on 32019 TKA confirms the higher revision risk in patients <65 at surgery [26].

In conclusion, we reported good (92.4 %) survivorship in a group of 245 Genesis TKA at 15–17 years’ follow-up and superior (98.3 %) survival of the Genesis II design. Revision risk was significantly increased with younger age (<69 years) at surgery and with PE thickness >11 mm, possibly related to longer shelf life in less frequently used insert sizes.
